# Creative interactions with data: using visual and metaphorical devices in repeated focus groups

**DOI:** 10.1177/1468794114557993

**Published:** 2016-02

**Authors:** Melanie Nind, Hilra Vinha

**Affiliations:** University of Southampton, UK; University of Southampton, UK

**Keywords:** focus groups, inclusive research, I-poems, learning disabilities, metaphor, participatory research, stimulus materials, visual methods

## Abstract

This article presents some of the emergent methods developed to fit a study of quality in inclusive research with people with learning disabilities. It addresses (i) the ways in which the methodology was a response to the need for constructive, transformative dialogue through use of repeated focus groups in a design interspersing dialogic and reflective spaces; and (ii) how stimulus materials for the focus groups involved imaginative and creative interactions with data. Particular innovations in the blending of narrative and thematic analyses and data generation and analysis processes are explored, specifically the creative use of metaphor as stimulus and the playful adaptation of I-poems from the Listening Guide approach as writing and performance. In reflecting on these methodological turns we also reflect on creativity as an interpretive lens. The paper is an invitation for further methodological dialogue and development.

## Introduction

It is not unusual for qualitative researchers to creatively develop and try out new and adapted techniques, methods and approaches as they explore, make sense of, and re-present complex social research phenomena (Coffey, 2011). Indeed methodological innovation is encouraged by a range of social contextual factors (Wiles et al., 2013; Xenitidou and Gilbert, 2012) including ethical concerns (Nind et al., 2013). While for some (such as Coffey, 2011) innovation is crucial and for others (e.g. Travers, 2009) it brings the dangers of poor research and fads, new research problems may demand solutions that push researchers to, if not outright innovate, at least experiment with new potentials for methods developed for purpose. Wiles et al. (2013: 19) refer to ‘the need to develop and experiment with methodological innovations that have the potential to address new and emerging research questions’, alongside ‘demonstrating and persuading the broader social science community of the affordance and credibility of new methods in relation to other existing methods’. Moreover, these authors highlight the pressures to disseminate innovations before they have been rigorously critiqued and allowed to mature into something robust. In this paper we discuss our own experimentation with method/ology, which was in part driven by need and in part driven by a desire to follow Coffey and Atkinson’s (1996) advice, and Edwards and Weller’s (2012) example, to be playful with analytic methods and angles. The intention is to kindle discussion of the potential affordances of this kind of design and these kinds of adaptations of emergent methods so that they might be developed further if they are useful to others, or perhaps stimulate further critique.

The paper is organised such that we first present the research context and the aims and needs of the study in question. We then discuss the design developed for the study and go on to the particular methods as part of this, discussing their rationale, roles and potential.

## The research context

The context for the methodological exploration discussed in this article was a study funded by the Economic and Social Research Council in the UK, which was aimed at building knowledge and capacity in inclusive research with people with learning disabilities (Nind and Vinha, 2012, 2014). In the process of securing funding and before recruiting a research fellow for the project Melanie had proposed to meet this aim by taking stock of the knowledge base, producing guidance on the issues and challenges, developing materials and case studies, and producing criteria for quality in inclusive research. Taking stock of the knowledge base and understanding quality in inclusive research though, were particularly complex and sensitive tasks demanding a creative response.

The term *inclusive research* in the field of learning disabilities was adopted by Walmsley and Johnson (2003) to allow for the continuity and reciprocity between participatory research – actively involving people with learning disabilities in decision-making and conduct of research – and emancipatory research – which is under full control of people with learning disabilities and in their interests. It was also intended to be a more accessible term and a more flexible concept, free of some of the dogma attached to these other concepts. Nonetheless, Walmsley and Johnson (2003: 16) are explicit about the principles on which inclusive research is based: that it ‘must address issues which really matter to people with learning disabilities, and which ultimately leads to improved lives for them’, ‘must access and represent their views and experiences’, and reflect ‘that people with learning disabilities need to be treated with respect by the research community’. This approach to research is a response to the desire to address the past wrongs of research that regarded people with learning disabilities as having nothing worthwhile to say, thereby making them at best ‘passive recipients of well-intentioned academics … and, at worst, dehumanized objects whose actions were observed and counted with no recognition of their humanity’ (Walmsley and Johnson, 2003: 11). Discussing people’s moves into inclusive research and what makes it good inclusive research, therefore, involved entering heated political territory.

The original motivation in seeking funding to conduct the research related to Melanie’s intellectual curiosity, both as a methodologist and as someone whose substantive research concerns have been in the realms of learning disability, social interaction and inclusion. The moral case for inclusive research with people with learning disabilities she found compelling. Yet she also understood from experience that research can be valid and emancipatory without always being inclusive (see also Danieli and Woodhams, 2005), and therefore inclusive research required better understanding. While some commissioners of research (particularly Joseph Rowntree Foundation and Heritage Lottery Fund in the UK) and much of the learning disability research community are convinced about the value of inclusive research, it is still rarely regarded as producing the best evidence – more often positioned alongside ‘real’, quality research – a necessary adjunct. The rhetoric around inclusive research has also reflected a ‘failure to grapple honestly’ (Walmsley and Johnson, 2003: 16) with the most sensitive questions about the challenges, and this needs to be recognised for investment in inclusive research to be wise investment. Some of Melanie’s research had been more inclusive, some less so, and she wanted to be in dialogue with other researchers, both those with learning disability labels and those with academic positions, who could reflect on a wider range of experiences of inclusive research.

It was evident that the taking stock could not be done without problematising something that was prone to being glorified, though constructive questions had been raised about whether the primary issue is the quality of the participation or the quality of the research (Greene, 2009; Freeman and Mathison, 2009), whether participatory research is necessarily ‘ethically or morally superior’ or ‘more enabling’ (Holland et al., 2008: 1), or whether the participation of people with learning disabilities is always meaningful and genuine (Aspis, 2000). It was timely, therefore, to try to un-stifle the debate and ‘challenge certain orthodoxies and assumptions in order to clarify what inclusive research is and how and where it can be applied’ (Walmsley and Johnson, 2003: 12). This meant accepting the argument of Grant and Ramcharan (2007) that inclusive research had come to the end of an initial phase in which practical knowledge had been gained, and it would be useful to agree the elements of that knowledge and enter a new phase that would include asking ‘how the knowledge claims of inclusive research can be assessed and authenticated’ and ‘whether good science and good inclusive research practice can be brought together’ (2007: 12).

## Adventures with focus group design

The rationale for choosing focus groups as the essence of the research design related to a desire to create vibrant interactive spaces in which best use could be made of constructive friction within the field. This, in turn, was influenced by reading of Freire (1970) and the methodological translation endeavoured by Hilra (Vinha, 2011) in her doctoral study, which embedded the Freirean dialectic of the praxis (action-reflection) in a continuous reflexive movement. A crucial idea was that facilitating dialogue would not just unveil reality but allow those communicating their reality to know it critically and to engage in re-creating knowledge in a communal way, transforming their understandings of themselves and their work. In challenging certain orthodoxies that had grown up we needed to challenge the concept of any actor in the research being an expert knower (based on academic knowing or the expert by experience idea); instead we sought to position everyone as ‘emergent becomings’, ‘always-unfinished subjects-in-the-making’, never ‘fully knowing, competent and rational’ (Gallacher and Gallagher, 2008: 511). In this way we could all learn and benefit from focus group dialogue. Morgan, Fellows and Guevara (2008) refer to the relatively short history of the use of focus groups in social science research, the proliferation in use of them, and some emergent alternatives to traditional focus group approaches. In face-to-face focus groups, they argue, there is ‘a self-conscious movement beyond the minimal goal of producing the kind of interaction that has manifest value as data for social science research’ (2008: 191). It was interaction we needed, but this came with considerable challenges because of the sensitivities associated with power dynamics in inclusive research. Focus groups offered an obvious route for interaction, but were potentially fraught.

The aim was to involve a range of inclusive researchers and for the research process to be dialogical – listening to and engaging with a range of researcher voices, reflective – embracing the praxis of naming the world collaboratively, and transformative – re-locating authority away from the individual researcher or researched and instead embedding it in the interactive space between them. This meant the need for dialogic spaces to be safe enough, but not too safe. Following StPierre’s (2009) argument that thoughts as well as voices constitute data, the dialogic spaces needed to be interspersed with reflective spaces. In the initial planning Melanie was drawn to adapting Hilra’s (Vinha, 2011) dialogic inquiry cycles and to using Freire’s (1970) concept of dialogue as creative and liberating. There was a need to ‘design for emergence’ (Morgan et al., 2008: 191) so that the research process was not imposed on the dialogic partners but responsive to them. We might have managed this within focus groups by structuring open-ended questions either side of specific focus topics, but this would not have given any of us the space to reflect and to transform our thinking. We therefore needed a repeated or reconvened focus group design (Morgan et al., 2008) in which participants could bring thinking done in and since previous dialogic encounters into later focus groups, enriching the dialogue and giving them more control. The reflective spaces between a series of focus group encounters would also allow time for the creation of stimulus materials to bring to each focus group encounter that could in some way enable ideas to flow between focus groups.

The repeated focus group design seemed to answer the call for a methodology in which inclusive researchers could engage in ‘deliberative, dialogic and democratic practice’ (Kamberelis and Dimitriadis, 2005: 887). As Bagnoli and Clark (2010: 103) argue, such focus groups can represent a non-hierarchical participatory approach, which is ‘more than just an exercise in capacity building or the production of “relevant” research … it also produces alternative knowledge and more effective ways of understanding complex situations and relationships’. The question remained, though, as to how the focus groups could un-stifle debate and talk about things that were difficult to talk about (e.g. whether there are limits to what inclusive research can achieve, or to who can be included in it, and how it maps on to other quality criteria in research). The answer came in developing the strategy of Madriz (2000) and Haw (2010) when addressing similar political and pragmatic challenges about who can speak safely with whom and about what in research with marginalised groups. This meant, somewhat riskily, rejecting a growing convention in inclusive research that everything in the research had to be done together and instead bringing together separate groups of inclusive researchers who were relatively homogenous in their relationship to research and who might feel able to talk openly together: a group comprising researchers with learning disabilities who led and conducted their own research, a group of researchers with and without learning disabilities who worked together collaboratively as co-researchers, and a group of academic researchers who used participatory approaches to actively engage people with learning disabilities in research. For the purposes of emergent design and responsive, transformative dialogue, these groups needed to have stable membership and build trust over time (three encounters each), so that they could engage, and re-engage, with ideas from their own and each other’s dialogic encounters. To add another perspective a single focus group of funders and policy maker supporters of inclusive research was added. The methodological emphasis was on non-judgmental group interviews where those with a common interest could share thinking without needing to reach consensus (Krueger and Casey, 2009).

The design was for the main focus groups to meet on three occasions in a sequence of the encounters allowing each group of insight-rich participants to take a turn in setting the agenda informing the questioning route for the other groups. Focus groups were to last two hours and the questioning route was structured conversationally from the initial invitation to start talking and share experiences into transitional and key open-ended questions of what makes inclusive research inclusive, challenging, possible, and of high quality. Ending questions were designed to promote considered reflection on the discussions and to ensure critical concerns had been voiced. The dialogic and reflective process was to culminate in a plenary event in which the different focus groups would join to discuss the key themes arising from the research. This reinforced the inbuilt ongoing validation process in which ideas were revisited, and it allowed for a final discussion about the meaning we had given to the participant-researchers’ discussions so that the most salient features could be checked and further built upon.

Inevitably the reality of the iterative focus groups was messier than the design implied. The planned sequence of encounters was interrupted by practical problems with agreeing meeting dates. Even being flexible with dates, membership was only about two-thirds stable across encounters and the size of groups fluctuated. To ensure we heard sufficiently from learning disabled researchers and to facilitate ease of travel we included an additional group of learning disabled researchers and their supporters. It may be that the preparatory work with them was insufficient, however, as there were tensions in the first encounter of this group in which some of the participant-researchers and supporters angrily asserted their authoritative knowledge about making ideas and consent forms accessible and research inclusive. Trust and rapport were lacking amongst those more ready to speak than to listen. A small group worked between meetings to provide feedback on how the focus group could work better and indeed a stronger sense of dialogue was achieved next time. Other groups were dialogic from the start, evident in the transcript statements such as ‘I understand, I agree with you’, ‘yes, I always felt that’, ‘I really enjoyed listening to …’, ‘I was thinking while you were talking’ and ‘I thought that was a good metaphor’.

## The role of stimulus materials

Morgan et al. (2008) argue that, compared with other fields, such as market research, social science researchers rely heavily on pure discussion of interview questions. The alternative they suggest is to use more stimulus materials – pictures, stories and so on – as a basis for discussion. Stimulus materials are created outside of the focus group and introduced by the moderator or created more emergently (as in their concept mapping example) generated by the group. Melanie was led to incorporate stimulus materials in the original design for two key reasons. First, we would be involving people with learning disabilities who might find tangible materials aided their expression and understanding of ideas. Moving away from pure talk might help to facilitate their active engagement in discussion. This took on further importance when Hilra became involved in making the design a lived reality as her earlier methodological work with (learning disabled) children (Vinha, 2011) had been underpinned by verbal and visual metaphor, through which she had created enabling spaces for dialogue with her participants. Second, the research necessitated exploring ways of allowing for different perspectives to be heard and shaped in interaction with others. It was important that the ideas discussed in the focus groups remained somewhat fluid so that participants did not feel driven to defend their positions, making them entrenched. In our taking stock of inclusive research we wanted to be able to communicate its complexity and diversity at a moment in history, rather than fix it. For the repeated, stratified focus group design to be effective it was necessary for ideas to flow across and between focus groups without making the discussions feel too repetitive. For this reason, the questioning routes for each group needed to communicate something of the interim analysis, something that communicated effectively while not being too polished, and this required stimulus materials.

Three main types of stimulus materials were used. The most straightforward and therefore not extensively discussed further here was of the concept mapping type and used in the single occasion focus group of funders and policy makers. There were two lots of stimuli incorporated into the questioning route: one was an oral introduction about emergent themes in the research so far, by a researcher with learning disabilities and her co-researcher and supporter (Lou Townson and Chloe Chapman-Brown),^1^ offering their perspective. This was politically important in the spirit of *nothing about us without us*; it meant that the idea of including people with learning disabilities in the conduct of research was not being discussed in the absence of anyone with learning disabilities or the ideas of researchers with learning disabilities. This desire to set a respectful tone was in tension somewhat with the desire for safe spaces and discussion that was not stifled; many of the decisions related to participatory or inclusive research can be about trade-offs (Edwards and Alexander, 2011). Nonetheless, the introduction made the focus group feel alive with important issues and critical and reflective engagement was therefore high. The other stimulus was the things about inclusive research that individuals in the group thought were important, which they were asked to write on cards at the start and add to if they so wished. The concept mapping, which happened later in the discussion involved physically organising these named elements into a diamond ranking. The diamond visual structure for this kind of exercise supports thinking about the hierarchy of concepts and generates dialogue about the complexities and nuances (Wall et al., 2013). The other types of stimulus materials involved more experimentation with adapting ideas from research for new purposes. First we discuss the use of a playful metaphor as stimulus, and second the use of a kind of I-poem idea taken from the Listening Guide approach (Gilligan et al., 2003).

## Metaphor: if inclusive research was a cake …

**Figure 1.**
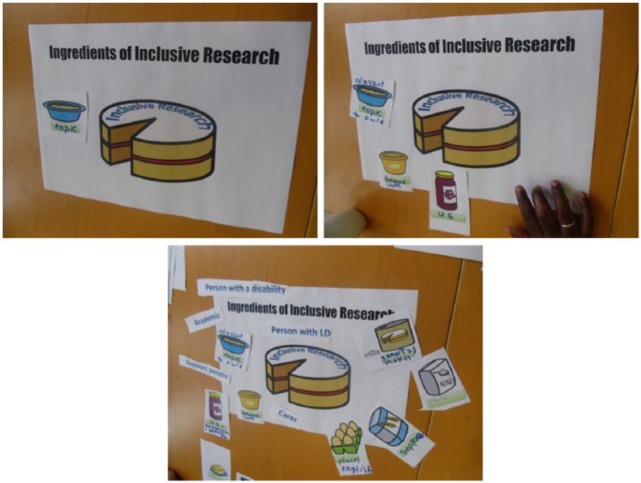
The cake metaphor.

Metaphors are accepted foci for qualitative researchers, but usually those used in everyday language or those used as a feature of thought in interpretation and theory-building (Todd and Harrison, 2008), such as Denzin and Lincoln’s (2008) bricoleur or quilt maker and Janesick’s (2003) choreography. Metaphor involves referring to, or understanding, ‘one kind of thing by means of another, thereby highlighting possible new aspects of a kind’ (Kvale and Brinkman, 2009: 48). In conceptual metaphors ‘the abstract notion [for example] of life (the target domain) is explained through the more concrete physical experience of [for example] a journey (the source domain)’ (Todd and Harrison, 2008: 481). The stimulus material involved exploring the target domain of inclusive research through the more concrete source domain of a cake. Indeed participant-researchers played around with ideas about the ingredients of the cake (inclusive research) and what made the cake good (quality in inclusive research). The first focus group to use this stimulus material, which involved both the idea and tangible visual materials representing a cake and its ingredients (see Figure 1) were the group of researchers with learning disabilities and their supporters and this was their second of three encounters. The physical products of their manipulation and labelling of ingredients were photographed to create stimulus materials for the focus group that met next, a different group of learning disabled and academic researchers who worked collaboratively. This, then, was a manifestation of our ‘serious play’ (Gauntlett, 2007) with techniques within the overall design.

The outcome of introducing the cake metaphor stimulus was some very rich dialogue. One participant-researcher in particular, Carl Bridden, was enthused by the idea and particularly liberated to think with this conceptual tool. In manipulating the visual pieces, he commented, ‘The jam is going to be us, because we’re on the middle of the cake’. The technique triggered a whole list of ingredients of inclusive research (a topic, us/people, background work, translators, money), but also some playfulness with ideas. When Hilra asked ‘who makes up the “us”?’, Durbali Roy responded ‘The jam factory!’ When Hilra clarified ‘Who are the people?’, Becca Cooper replied ‘strawberries, raspberries!’ When we discussed academics as possible ingredients and Melanie asked, ‘Are you happy that they are in this cake?’ Becca replied ‘you are university jam’. The enthusiasm amongst the learning disabled participant-researchers was matched by, perhaps aided by, the enthusiasm of those there to support them. When asked about the recipe for bad inclusive research, one supporter (Julie Davies) leapt on the metaphorical idea responding, ‘It would be all filling and no substance’. As well as making verbal contributions people were moving the images, labelling with pens and thinking. As moderators we were both in the moment and reflecting on the moment: the transcript records a check ‘Are we going too fast, Michael?’ and response ‘I’m thinking’ and we recall evaluating then and afterwards the ability of the stimulus materials to provoke and yet still to fail to act as a leveller when people process information at different speeds and in different ways.

The focus group members discussing the metaphor somewhat second hand were discussing other people’s ideas generated by the stimulus. Nonetheless, they took the original metaphor stimulus, in interaction with the outcomes from it to date, and breathed new life into it. They generated their own conceptual metaphors, talking about the different types of cakes (inclusive research) that could be made depending on how the ingredients were put together, about commissioned research as ‘factory made’ and life story and partnership research being more ‘home made’ (Ian Buchanan). Chloe Brownlee-Chapman introduced the metaphor of ‘sprinkles’ used as decorative elements and the ‘baking tray’ as the academic researcher who ‘holds things together’. Jan Walmsely added another dimension: ‘we need to think about what we say to funders about the taste of this cake that is superior to a cake that is made without the inclusive ingredient’. Melanie Chapman added the idea of the cake turning out differently to how you and the funders expected it to be. The metaphor then, was an effective stimulus for deep thinking and for interaction between ideas.

## Iterative data analysis: playing with I-poems

Informing the choice of stimulus materials and their use was the importance of themes arising from earlier discussions providing a focus for our talk, but equally, the importance of such themes not overpowering the individual and collective narratives or working against fresh ideas. The ongoing iterative analysis in the reflective spaces between the dialogic spaces, therefore, had complex layers of work to do in shaping the stimulus materials and questioning routes as the research progressed. This meant using a ‘range of interconnected interpretive practices’ to ‘get a better understanding’, each practice making ‘the world visible in a different way’ (Denzin and Lincoln, 2005: 4). The analysis comprised a process of coding themes alongside identifying core narratives. The former was about ‘bring[ing] order to experience by seeing individual things as belonging to a category’ (Polkinghorne, 1995: 10) while interest in the narratives was in their power to describe ‘what happened’ intermingled with ‘emotions, thoughts and interpretations’ (Chase, 2005: 656) and in not reducing people’s lives and stories to data in an objectifying way. Thematic analysis involved assigning codes (thematic labels) (e.g. *ways of working together, research topics, outcomes*) and sub-codes (e.g. *outcomes/impact, outcomes/data, outcomes/report, outcomes/DVD*) to parts of the transcripts. The more detailed sub-codes were later grouped into more coherent groups (e.g. *outcome impact, outcome process, outcome product format*). In the interpretative process some codes were assigned greater importance and others were merely logged for transparency. The themes culminated in units of meaning that were checked in the final plenary focus group. A less standard approach was applied to the narrative part of the analysis.

In common with narrative researchers we were viewing ‘narratives as verbal action – as doing or accomplishing something’ (Chase, 2005: 657) such as explaining, informing, confirming or challenging. For the narrative analysis we worked two processes simultaneously; one was highlighting the statements made in the first person (e.g. *‘I’m in the Work in Progress team’, ‘I want to make a difference’, ‘I’ve seen more exciting things’, ‘I look back’, ‘I wish I had’*) and the other was assigning a narrative code identifying what the narrative was about or what it was for (e.g. *narratives about belonging, narratives about motivation, witnessing narratives, reflecting narratives*). This working of the transcript data felt important in terms of being true to what was being said and to the dialogic process. It was less clear, initially, how the narrative analysis would be used.

The idea of coding the first person statements came from the literature on the voice-centred relational method/Listening Guide (Brown and Gilligan, 1992) and specifically the parts about I-poems (Edwards and Weller, 2012; Gilligan et al., 2003). The Listening Guide is an approach to analysis of narrative data that counters the implicit assumption that ‘a person’s “story” is singular and transparent’ (Sorsoli and Tolman, 2008: 495) and instead seeks to listen to the multiple voices threaded through, thus using multiple interpretive readings to get at the underlying complexity. The approach was developed for more psychodynamic work and for working with the transcripts of individual interviews, neither of which related to this context. Nonetheless the concepts of plot and voice were interesting in the light of the repeated references the participant-researchers in this study made to their identities, learning journeys, experiences and hopes. Gilligan and colleagues use the concept of contrapuntal voices – which are distinct and working with and against each other to infuse meaning – and this echoed through the reading of the focus group transcripts which concerned dynamics and story form as well as content.

The construction of I-poems in the Listening Guide process involves first, identifying parts narrated in the first person (or second person when linguistically it is used to represent oneself) with their immediately associated relevant text, and second, taking these sections out of their original context and into an unpunctuated exact order list. These are placed on separate lines to resemble a poem (Gilligan et al., 2003). The original purpose of constructing I-poems was analytical, and we had also focused on I-statements as a way of not losing the sense of individual voices amongst the noise of the concepts and leitmotifs. We were using them iteratively to build a picture, and thus they were shaping as well as being shaped by the dialogic process. For Edwards and Weller (2012: 203) an I-poem analysis ‘draws attention to the research subject’s subjectivity – how they understand and speak about themselves, it also produces a particular analytic ontology – how the researcher is placed in relation to the subject and their social reality’. Similarly, we valued this reflexive potential regarding the mix of emotional and intellectual responses and the dynamic of speaking and listening. Inclusive research is ultimately about the people we want to include and so people are important as well as ideas.

Smith (2013) constructed I-poems from interview and participant diary data, thus adapting the process. He, though, like Edwards and Weller (2012) retained use of Gilligan and colleagues’ four steps of sequential listening: listening to the plot (and the researcher’s response to it); constructing I-poems to really attend to the voices; listening for contrapuntal voices; and composing an analysis. We were much less focused on the rigorous attention to this analytic process and much more concerned with the emergent potential of I-poems in the iterative process of feeding ideas and stories across focus groups. These, could perhaps tell much about how people were story-ing their involvement with inclusive research and thus bring previous focus groups’ discussions alive in a new focus group context. This idea for an innovative use of I-poems was recorded in Melanie’s fieldnotes with the capital letters and explanation mark suggesting an ‘aha’ moment: ‘IDEA! Maybe use poems for Leeds and Manchester [focus groups]’. The idea then took on an important dimension in the design as a vehicle to communicate, across focus groups, key ideas and narratives using the participant-researchers’ own multiple voices to lay bare the multiple, interwoven ways of experiencing inclusive research and telling inclusive research.

Our use of I-poems had a relationship with inclusive research that was full of almost paradoxical tensions. It felt democratic that ideas would be presented in participant-researchers’ exact words and narrating their experiences of inclusive research offered promise of transformation. Yet the poems were shaped by our (Melanie and Hilra’s) analysis and construction and the power we held in the research. The words were not raw data but situated, textual, representing a certain kind of telling (Silverman, 1993). Our researcher voices were neither authoritative nor muted but more interactive in Chase’s (2005) terms. The I-poems were less ‘gazing at’ the participants and more ‘standing alongside’ them (Edwards and Weller, 2012: 215), yet still interpreting their (and our) social realities. Byrne et al. (2009) saw the participatory research potential of the Listening Guide and used the clear steps to make the analysis process accessible to the early school leavers they were involving as researchers, but this was not our goal. We were more concerned with a new use for I-poems as co-constructed products with a stimulatory role in the ongoing cyclical process of dialogue. By working from focus group transcripts, instead of one person’s multiple voices being teased out it was as if the voices of multiple participants became one. We were always cautious about writing about the participant-researchers, doing research *on* them, but it was inclusive research and not people that was the primary object of inquiry. Like StPierre’s older women involved in her research on reflexivity, these people were present in the texts, everywhere in the analysis, ‘provocateurs’ of fresh ideas and like her we did not want to ‘be unworthy’ of them (Richardson and StPierre, 2005: 973).

The new function for the I-poems demanded some poetic license be taken with the Listening Guide process. There was an innovative dimension in that the poems would need to be read aloud, in part because not all the participant-researchers would be able to read them for themselves, but more because this would mean the stimulus material was experienced as a group ‘*experience of consumption*’ (Leavy, 2008: 344; original emphasis). There was therefore an element of performance to consider, though this was not the first performance, the narratives themselves being ‘socially situated interactive performances’ of ‘the self, experience, and reality’ (Chase, 2005: 657). The I-statements were identified and grouped under themes, for example:

***Who we are***

I’m a co-researcher

I’m from People First

I’m a parent with a learning disability

I’m a Lecturer at the Open University

I’m a part-time research student

I’ve been working… for a very long time

I am Lisa

This is me and the research I’ve done

I am good at speaking up

and

***Our beginnings***

It started back in 2002

I spent the first 15 years of my working life

I’d never done a project myself

My first days

I didn’t know anything

I started looking at history

From asking questions I got into research

I’d always had a belief

I heard the lottery were funding

I went off to do a PhD

I ended up going to Helsinki

We were breaking with Listening Guide rules in doing this thematic re-organising of I-statements and then in not presenting them in the order in which they appeared, but as Melanie reflected: ‘I was the narrator now, it was I who was creating the sense of order in the material. I was also adding stanzas as part of the creative process, making the poems more coherent and appealing to the ear’:

***Our journeys***

I started off

I ended up

I didn’t intend to be a researcher

I was working with

I got involved

I joined the research team

I learnt something

I started asking loads of questions

I decided

I think I stumbled into inclusive research by making mistakes

As the I-poem dimension of audience took hold then so did the dimension of writing as a method of knowing. Richardson and StPierre (2005: 959) maintain that qualitative researchers have latterly found ‘writing as a method of inquiry to be a viable way in which to learn about themselves and their research topic’. In experimenting with writing data as ‘a poetic representation’ we were ‘using writing as a method of knowing’ (Richardson and StPierre, 2005: 974), one of the explorations Richardson recommends. This was, as Brady (2005) discusses, complementing rather than replacing other ways of knowing. Even though the words did not come from us, writing – or composing – the I-poems gave a new perspective on the data. Writing being research fits with a core idea in qualitative research of the researcher being the research instrument making this more comfortable than it might have been.

In selecting and reorganising some of the phrases from the transcripts we could be said, in the poststructural reading of Richardson and StPierre (2005), to be creating a new form of narrative writing, less reflecting the focus groups’ social realities and more interpreting them to create new meaning. Just as the participant-researchers’ meaning was ‘dependent on the discourses available to them’ (Richardson and StPierre, 2005: 961) so these new narrative poems were located in the cultural norms of self-advocacy and research as a site of politically sensitive struggle. Yet they could play a role in the desired process of helping us all to know inclusive research afresh, and perhaps also to ‘understand ourselves reflexively as persons writing from particular positions at specific times’ (Richardson and StPierre, 2005: 962). For example:

***Our reflections***

I look back

I always remember

I thought at the time

I didn’t say it

I wish I had

I wish you had too

I was entirely wrong

People have accused me

It made me think

I surprise myself

I’ve realised that

I learnt a lot about

We are still learning

StPierre reflects, ‘for me, writing *is* thinking, writing *is* analysis, writing *is* indeed a seductive and tangled method of inquiry’ (Richardson and StPierre, 2005: 967; original emphasis). She argues that ‘language cannot serve as a transparent medium that mirrors, “represents”, and contains the world’ (2005: 968); it doesn’t ‘simply transport meaning’ (2005: 968) so that we might work towards consensus. Thus ‘interpretation is not the discovery of meaning in the world but rather the “introduction of meaning” (Spivak, 1974, p.xxiii)’ (2005: 968), ‘the interpreter has to assume the burden of meaning-making, which is no longer a neutral activity of expression that simply matches word to world’ (2005: 968–9).

Ultimately the I-poems brought creative and analytical processes closer together, intertwining process and product as Richardson and StPierre (2005) describe. By ‘performing’ the poems at the start of a focus group we understood that they were being judged for their aesthetic merit and emotional impact as well as their ability to re-present our discussions. Some responses to hearing the poems were about the form itself. Lou Townson (self-advocate researcher) responded: ‘What a good idea of [.], what a good way of doing it. Sometimes, if you do transcripts [they] can be a bit long, but actually putting like that condenses everything.’ She then added warm approval: ‘I like that, it’s an idea that I never thought about, but, I like that’. Malcolm Eardley, another self-advocate researcher, made an effortful response to sum up the nature of the thing and its impact: ‘That bit it’s just like a story. It’s very [.]. When they get … writing like this, in put[ting] word[s] together as a poem, putting words together, what happens and what they are doing, it’s just getting the feeling of … the poems together’. In another focus group, Dubarli Roy, a learning disabled researcher commented on the lyrical aesthetics: ‘I quite liked it, because feel all we went, you got a voice as you have a voice, and you want to sing a song. Sometimes you want to learn how to sing a song, you want to know how to do poems, and it’s really a good choice.’ Gareth, in contrast, observed ‘They’re not Pam Ayres’!

A performance exists primarily in the live moment, and its ephemeral quality (Leavy, 2008) adds a certain charge. The following (narrative but less of an I-poem) triggered a particular emotional response, a sighed, spoken, echoing of the sense of relief at the end.

***Our experiences with funders***

It [our bid] was kept hanging

We did ring them

The funders wouldn’t give us the money

We kept going back to them

They don’t really give you reasons

It got to the final stage

They were so impressed they actually gave us funding

The creative variation on I-poems then, shifted from offering a scientific analytical lens, to combining scientific and creative lenses, which Richardson and StPierre (2005: 964) might see as a ‘“social science art form” – a radically interpretive form of representation’ allowing us to ‘see more deeply’. As Leavy (2008: 344) argues, in the doing of performance ‘meaning is constructed and multiplied’, negotiated with the audience with social science and emancipatory potential not yet fully understood. In constructing and reading the poems Melanie was in some small way embodying the data (see Leavy, 2008). Indeed the other stimulus materials – the co-produced ‘cakes’ might be seen in this light also. While Richardson and StPierre see thinking as happening in the writing, our thinking was happening in the talking, creating, listening and writing.

## Conclusion

The whole movement toward inclusive research is about values, ideas, politics. This forms a kind of ‘methods gap’ (Hesse-Biber and Leavy, 2008; Leavy, 2008) in which abstract theory places demands on methodological development. In researching inclusive research we sought to create a dynamic, dialogic process. In doing so we came to understand the subject of our inquiry – inclusive research – in relation to trust but also risk, to social responsibility but also playfulness. Ultimately we transgressed some of the orthodoxies of the inclusive research genre, just as we transgressed or adapted aspects of the Listening Guide. We gave ourselves permission to engage in our own methodological development, engaging as much with ideas from the cutting edge of qualitative research as with ideas from inclusive research to compose an approach making use of, but not being constrained by, principles from each. The stimulus materials, metaphor and I-poems worked within the iterative focus group design where their potential for provoking and reflecting transformative dialogue was realised. Responses to them were authentic in the way that their use was authentic; the methods were included for a carefully considered purpose and engagement with them was in this spirit. They could not make everything accessible to everyone all of the time, but they could give us a different purchase on the topic of inclusive research and the analysis of knowledge in the making.

Taking risks, facing our own epistemological doubts, and daring to listen fully to everyone involved made us aware of our moral obligation to challenge ourselves to further the discussion, to invite other researchers to dig deep into the contradictions and complexities of doing research inclusively rather than pursue a fixed methodology with the label of inclusive research. We need to reflect on how we are ‘producing’ (StPierre, 2009: 230) ourselves in interaction with the labels and descriptions assigned to us. Extending this substantive and methodological dialogue has been the goal in writing the paper. Our desires to connect inclusive research with wider research developments and to treat ourselves as emergent instruments of research and crafters of research methods are genuine. Our claims to innovation and to inclusion may be modest but we have been ambitious in our attempt to do something creative and constructive with the tensions before us.

## Notes

1. Participant-researchers in this study preferred to be named and credited for their contribution rather than be anonymised.

## References

[bibr1-1468794114557993] Aspis S (2000) Researching our own history: who is in charge? In: Brigham L (eds) Crossing Boundaries: Change and Continuity in the History of Learning Disabilities. Kidderminster: BILD Publications, 1–6.

[bibr2-1468794114557993] Bagnoli A Clark A (2010) Focus groups with young people: a participatory approach to research planning. Journal of Youth Studies 13(1): 101–119.

[bibr3-1468794114557993] Brady I (2005) Poetics for a planet: discourse on some problems of being-in-place. In: Denzin NK Lincoln YS (eds) The Sage Handbook of Qualitative Research (3rd ed.). Thousand Oaks, CA: Sage, 979–1026.

[bibr4-1468794114557993] Brown LM Gilligan C (1992) Meeting at the Crossroads: Women’s psychology and girls’ development. Cambridge, MA: Harvard University Press.

[bibr5-1468794114557993] Byrne A Canavan J Millar M (2009) Participatory research and the voice-centred relational method of data analysis: is it worth it? International Journal of Social Research Methodology 12(1): 67–77.

[bibr6-1468794114557993] Chase SE (2005) Narrative inquiry: multiple lenses, approaches, voices. In: Denzin NK Lincoln YS (eds) The Sage Handbook of Qualitative Research (3rd ed.). Thousand Oaks, CA: Sage, 651–679.

[bibr7-1468794114557993] Coffey A (2011) Revisiting innovation in qualitative research. Qualitative Researcher 13: 1–2.

[bibr8-1468794114557993] Coffey A Atkinson P (1996) Making Sense of Qualitative Data: Complementary Research Strategies. London: Sage.

[bibr9-1468794114557993] Danieli A Woodhams C (2005) Emancipatory research methodology and disability: a critique. International Journal of Social Research Methodology 8(4): 281–296.

[bibr10-1468794114557993] Denzin NK Lincoln YS (eds) (2005) The discipline and practice of qualitative research. In: The Sage Handbook of Qualitative Research (3rd ed.). Thousand Oaks, CA: Sage, 1–32.

[bibr11-1468794114557993] Denzin NK Lincoln YS (2008) Introduction: The discipline and practice of qualitative research. In: Denzin NK Lincoln YS (eds) Strategies of Qualitative Inquiry (3rd ed.). London: Sage, 1–34.

[bibr12-1468794114557993] Edwards R Alexander C (2011) Researching with peer/community researchers. In: Williams M Vogt WP (eds), The Sage Handbook of Innovation in Social Science Research Methods. London: Sage.

[bibr13-1468794114557993] Edwards S Weller S (2012) Shifting analytic ontology: using I-poems in qualitative longitudinal research. Qualitative Research 12(2): 202–217.

[bibr14-1468794114557993] Freeman M Mathison S (2009) Researching Children’s Experience. New York: Guilford Press.

[bibr15-1468794114557993] Freire P (1970) Pedagogy of the Oppressed. New York: Continuum.

[bibr16-1468794114557993] Gallacher L Gallagher M (2008) Methodological immaturity in childhood research? Thinking through ‘participatory methods’. Childhood 15(4): 499–516.

[bibr17-1468794114557993] Gauntlett D (2007) Creative Explorations: New Approaches to Identities and Audiences. London: Routledge.

[bibr18-1468794114557993] Gilligan C Spencer R Weinberg MK Bertsch T (2003) On the listening guide: A voice-centered relational method. In: Camic PM Rhodes JE Yardley L(eds) Qualitative Research in Psychology: Expanding Perspectives in Methodology and Design. Washington, DC: American Psychological Association, 157–172.

[bibr19-1468794114557993] Grant G Ramcharan P (2007) Valuing People and Research: The Learning Disability Research Initiative. Overview Report, Department of Health. Available at: http://www.hscbusiness.hscni.net/pdf/DH_Learning_disability_research_report-2007_pdf.pdf

[bibr20-1468794114557993] Greene S (2009) Accessing children’s perspectives and experience: some impediments. Paper presented at: Advancing Participatory Research Methods with Children and Young People NCRM/Child Well-Being Research Centre, London, 23 February 2009.

[bibr21-1468794114557993] Haw K (2010) Using video as a trigger: facilitating participation in research with hard to reach groups. Paper presented at: Potential & possibilities: using video stimulated recall and related methods in research University of Southampton, 28 January 2010.

[bibr22-1468794114557993] Hesse-Biber SN Leavy P (2008) Pushing on the methodological boundaries: the growing need for emergent methods within and across the disciplines. In: Hesse-Biber NS Leavy P (eds) Handbook of Emergent Methods. New York, Guilford Press, 1–15.

[bibr23-1468794114557993] Janesick VJ (2003) The choreography of qualitative research design: minuets, improvisations, and crystallization. In: Denzin NK Lincoln YS (eds) Strategies of Qualitative Inquiry (2nd ed.). London: Sage, 379–399.

[bibr24-1468794114557993] Kamberelis G Dimitriadis G (2005) Focus groups: Strategic articulations of pedagogy, politics and inquiry. In: Denzin NK Lincoln YS (eds) The Sage Handbook of Qualitative Research (3rd ed.). Thousand Oaks, CA: Sage, 887–907.

[bibr25-1468794114557993] Krueger RA Casey MA (2009) Focus Groups: A Practical Guide for Applied Research. Thousand Oaks, CA: Sage.

[bibr26-1468794114557993] Kvale S Brinkman S (2009) Interviews: Learning the Craft of Qualitative Research Interviewing. London: Sage.

[bibr27-1468794114557993] Leavy P (2008) Performance-based emergent methods. In: Hesse-Biber NS Leavy P (eds) Handbook of Emergent Methods. New York: Guilford Press, 343–357.

[bibr28-1468794114557993] Madriz E (2000) Focus groups in feminist research. In: Denzin NK Lincoln YS (eds) Handbook of Qualitative Research (2nd ed.). Thousand Oaks, CA: Sage, 835–850.

[bibr29-1468794114557993] Holland S Renold E Ross N Hillman A (2008) Rights, ‘right on’ or the right thing to do? A critical exploration of young people’s engagement in participative social work research. NCRM Working Paper Series 07/08. Available at: http://eprints.ncrm.ac.uk/460/

[bibr30-1468794114557993] Morgan D Fellows C Guevara H (2008) Emergent approaches to focus groups research. In: Hesse-Biber NS Leavy P (eds) Handbook of Emergent Methods. New York: Guilford Press, 189–205.

[bibr31-1468794114557993] Nind M Vinha H (2012) Doing research inclusively, doing research well? Report of the study: Quality and capacity in inclusive research with people with learning disabilities. University of Southampton Available at: http://www.southampton.ac.uk/education/research/projects/quality_and_capacity_in_inclusive_research_with_learning_disabilities.page

[bibr32-1468794114557993] Nind M Vinha H (2014) Doing research inclusively: bridges to multiple possibilities in inclusive research. British Journal of Learning Disabilities 42(2): 102–109. DOI:10.1111/bld.12013.

[bibr33-1468794114557993] Nind M Wiles RA Bengry-Howell A Grow GP (2013) Methodological innovation and research ethics: Forces in tension or forces in harmony? Qualitative Research 13(6): 650–667.

[bibr34-1468794114557993] Polkinghorne DE (1995) Narrative configuration in qualitative analysis. In Hatch JA Wisnieski R (eds) Life History and Narrative. London: Falmer Press, 5–23.

[bibr35-1468794114557993] Richardson L StPierre EA (2005) Writing: a method of inquiry. In: Denzin NK Lincoln YS (eds) The Sage Handbook of Qualitative Research (3rd ed.). Thousand Oaks, CA: Sage, 959–978.

[bibr36-1468794114557993] Silverman S (1993) Interpreting Qualitative Data: Methods for Analysing Talk, Text and Interaction. London: Sage.

[bibr37-1468794114557993] Smith D (2013) What does risk look like in an educational setting for children and young people with autism? Unpublished Masters dissertation, University of Southampton, UK.

[bibr38-1468794114557993] Sorsoli L Tolman DL (2008) Hearing voices: listening for multiplicity and movement in interview data. In: Hesse-Biber NS Leavy P (eds) Handbook of Emergent Methods. New York: Guilford Press, 495–523.

[bibr39-1468794114557993] Spivak GC (1974) Translator’s preface. In: Derrida J Of Grammatology (Trans. Spivak GC). Baltimore, MD: Johns Hopkins University Press, ix–xc.

[bibr40-1468794114557993] StPierre EA (2009) Afterward: decentering voice in qualitative inquiry. In: Jackson AY Mazzei LA (eds) Voice in Qualitative Inquiry: Challenging Conventional, Interpretive, and Critical Conceptions in Qualitative Research. Abingdon: Routledge.

[bibr41-1468794114557993] Todd Z Harrison SJ (2008) Metaphor analysis. In: Hesse-Biber NS Leavy P (eds) Handbook of Emergent Methods. New York: Guilford Press, 479–493.

[bibr42-1468794114557993] Travers M (2009) New methods, old problems: a sceptical view of innovation in qualitative research. Qualitative Research 9(2): 161–179.

[bibr43-1468794114557993] Vinha MHGL (2011) Learners’ perspectives of identity and difference: a narrative study on visual and verbal representation of self and other. PhD Thesis, University of Southampton, UK.

[bibr44-1468794114557993] Wall K Higgins S Hall E Woolner P (2013) ‘That’s not quite the way we see it’: the epistemological challenge of visual data. International Journal of Research & Method in Education 36(1): 3–22.

[bibr45-1468794114557993] Walmsley J Johnson K (2003) Inclusive Research with People with Learning Disabilities: Past, Present and Futures. London: Jessica Kingsley.

[bibr46-1468794114557993] Wiles RA Bengry-Howell A Nind M Crow G (2013) But is it innovation? The development of novel methodological approaches in qualitative research. Methodological Innovation Online 8(1): 18–33.

[bibr47-1468794114557993] Xenitidou M Gilbert N (2012) Introduction to the special issue: the processes of methodological innovation narrative accounts and reflections. Methodological Innovations Online 7(1): 1–6.

